# Dissemination of Class 1, 2, and 3 Integrons Among Multidrug‐Resistant Isolates of *Pseudomonas aeruginosa*

**DOI:** 10.1002/mbo3.70447

**Published:** 2026-11-02

**Authors:** Mahdi Hosseini Bafghi, Abbas Baghaie, Razieh Nazari

**Affiliations:** ^1^ Department of Laboratory Sciences, Faculty of Paramedical and Rehabilitation Sciences Mashhad University of Medical Sciences Mashhad Iran; ^2^ Department of Microbiology, Qo.C Islamic Azad University Qom Iran

**Keywords:** antibiotic resistance, gene cassette, integron, nosocomial infection, *Pseudomonas aeruginosa*

## Abstract

*Pseudomonas aeruginosa* is one of the most important opportunistic pathogens, with a central role in nosocomial infections. Bacterial antibiotic resistance caused by mutations in chromosomal genes, acquisition of plasmids, and related to integrons has been reported. This project aimed to investigate Class 1, 2, and 3, and gene cassettes in Class 1 integrons, among 187 clinical isolates of *P. aeruginosa* from several hospitals in central Iran, with confirmation by standard microbiological assays. Antibiotic resistance testing was performed on 18 antibiotics across different categories using the Kirby–Bauer method according to CLSI standards. Then, the presence of Class 1, 2, and 3 integrons on all isolates and the variable region in the strains containing Class 1 integron was evaluated using a polymerase chain reaction (PCR) assay. Sequenced PCR products by size and analyzed for the determination of gene‐cassette arrays. Among isolates, 73 (39.0%) and 14 (7.5%) contained Class 1 and 2 integrons, respectively, and a Class 3 integron was not observed. The amplicons of 800, 1700, and 2200 bp contained gene cassettes, including *aadB*, *blaOXA10‐aac(6′)‐II*, and *aadB‐blaOXA10‐aac(6′)‐Ib*. The Antibiotic resistance phenotype of gene‐cassette‐containing isolates revealed great conformity with the expected phenotype, which depends on the type of resistance genes. Considering the high prevalence of Class 1 and 2 integrons containing gene cassettes for beta‐lactamases and aminoglycoside‐modifying enzymes among resistant *P. aeruginosa* isolates, it is necessary to adopt appropriate strategies to control and treat hospital infections to prevent the spread of these genetic elements.

## Graphical abstract

Clinical *Pseudomonas aeruginosa* isolates showed widespread Class 1 and 2 integrons carrying diverse resistance‐associated gene cassettes, including *aadB*, *blaOXA10‐aac(6′)‐II*, and *aadB‐blaOXA10‐aac(6′)‐Ib*, which were strongly associated with antibiotic resistance phenotypes, highlighting the need for enhanced surveillance and targeted infection‐control strategies.

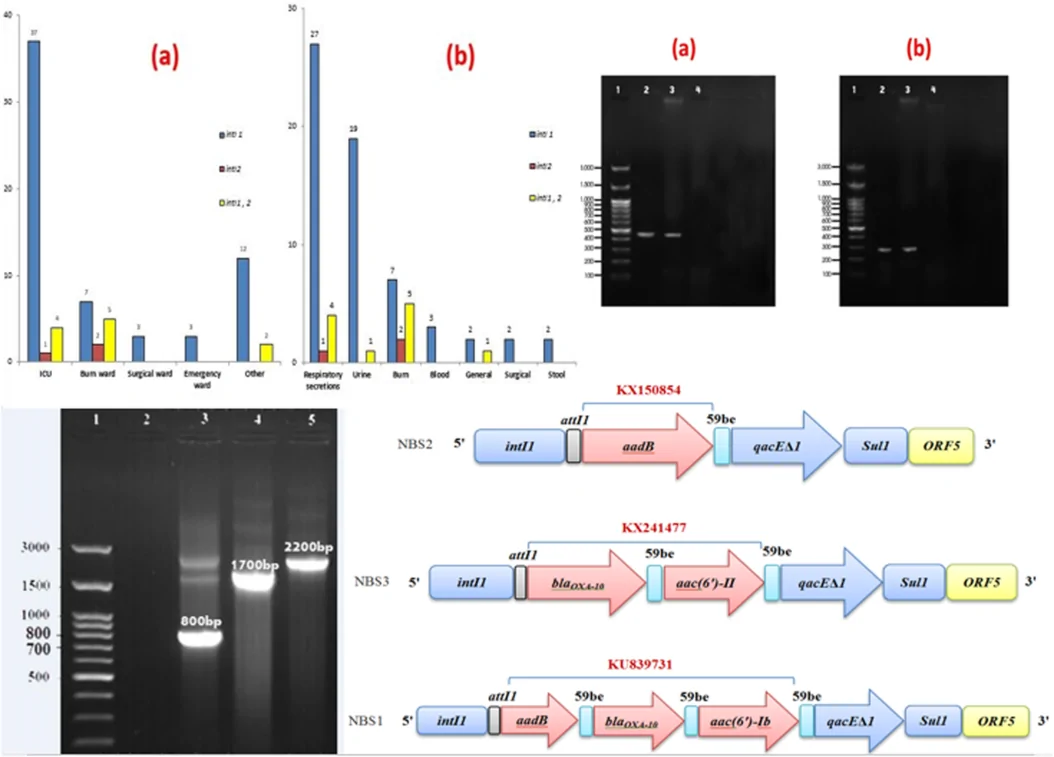

## 1 Introduction

Healthcare‐associated infections (HAIs), especially hospital‐acquired infections caused by a multitude of pathogens, including bacteria, fungi, and even viruses, are known as a significant contributor to the mortality rates in hospitalized patients (Liu and Dickter 2020). These infections disrupt the course of treatment and recovery, often leading to the development of severe complications during critical medical interventions (Boev and Kiss 2017; Mcgrath and Asmar 2011). Perhaps the most concerning aspect of nosocomial infections is the emergence of multidrug‐resistant (MDR) pathogens (Tahan et al. 2024). These resilient microbes have developed resistance to commonly used antibiotics, rendering treatment options limited and significantly more expensive (Zaragoza et al. 2020). The escalating threat of antibiotic resistance in bacteria necessitates continuous exploration of novel therapeutic strategies to combat infectious diseases and effectively safeguard patient well‐being (Ghanipour et al. 2023; Neshani et al. 2025).

The ubiquitous Gram‐negative bacterium *Pseudomonas aeruginosa* has become a notorious agent in HAIs, wreaking havoc in intensive care units and burn centers (Maldonado et al. 2016). *P. aeruginosa*'s capacity to inflict harm transcends its mere prevalence. This bacterium has very good antibiotic resistance mechanisms that make many frontline antibiotics essentially ineffective against it (Nanvazadeh et al. 2013; Zeraatkar, Tahan, Rostami, et al. 2023). Efflux pumps, enzymatic degradation of antibiotics, and a formidable outer membrane all contribute to *P. aeruginosa*'s ability to evade treatment. This multifaceted resistance not only complicates treatment regimens but also significantly increases treatment costs and mortality rates among vulnerable patient populations (Folic et al. 2021; Gonzalez et al. 2018). Understanding the intricate mechanisms employed by *P. aeruginosa* to thwart antibiotic treatment is paramount for developing effective strategies to combat this cunning pathogen and safeguard patient well‐being (Zeraatkar, Tahan, Sadeghian, et al. 2023). Therefore, this research addresses the specific role of integrons, a concerning genetic structure, and its potential contribution to *P. aeruginosa's* antibiotic resistance profile (Liao et al. 2022).

Integrons are genetic platforms capable of capturing, rearranging, and expressing gene cassettes, including antimicrobial resistance determinants, thereby facilitating the emergence and dissemination of MDR bacteria. (Goudarzi and Azimi 2017). Unlike plasmids, integrons themselves are not necessarily self‐replicating genetic elements; rather, they can occur within different genetic contexts. In clinical bacteria, particularly mobile Class 1 and 2 integrons, integron structures may be associated with plasmids, transposons, or the bacterial chromosome, providing an important mechanism for the acquisition and horizontal dissemination of resistance gene cassettes. This mobility and plasticity make integrons critical drivers in the widespread acquisition and expression of antibiotic‐resistance genes (ARGs) among clinical pathogens (Ghaly et al. 2020). These resistance genes are housed within mobile cassettes that can be shuffled and exchanged between integrons, enabling bacteria to rapidly evolve and adapt to various antibiotics (Munita and Arias 2016). Among the important variable genes that lead to this phenomenon, we can mention *aadB*, *blaOXA10‐aac(6′)‐II*, and *aadB‐blaOXA10‐aac(6′)‐Ib* (Kani and Alabdali 2023; An et al. 2023). This modular design of integrons, coupled with their presence on mobile genetic elements, poses a unique challenge and underscores the need for a deeper understanding of their role in antibiotic resistance.

Current research investigates the specific features of three classes of integrons (1–3) identified in *P. aeruginosa*, a bacterium that causes hospital‐acquired infections. Class 1 integrons stand out for their frequent presence in clinical isolates and their well‐established association with MDR phenotypes (Fadare et al. 2023). Their structure offers a potential target for therapeutic intervention. By inhibiting the *intI1* integrase gene, a key element responsible for capturing and integrating gene cassettes, or by exploring novel combination therapies, we might be able to disrupt integron activity and disarm these MDR pathogens (Bhat et al. 2023). Class 1 integrons possess a fascinating architecture. They harbor a specific attachment site (*attI1*) where gene cassettes can integrate using the *intI1* integrase. Additionally, a promoter (Pc) ensures the efficient expression of the resistance genes encoded within the cassettes. Flanking inverted repeat regions (*IRi* and *IRt*) serve as recognition sites for integrase during recombination (Wang et al. 2023; Brovedan et al. 2021). Understanding these intricate components is vital for devising strategies to weaken integrons and restore antibiotic effectiveness against *P. aeruginosa* and other MDR bacteria.

Class 2 integrons, recognized for the diversity of gene cassettes they harbor, are typically positioned proximal to the left end of nonreplicating transposons (Sabbagh et al. 2021). In contrast, Class 3 integrons, closely mirroring Class 1, exhibit a lower recombination frequency and orchestrate the excision and integration of cassettes at the *attI3* site (Deng et al. 2015).

The association between the resistance phenotype and gene cassettes located in the variable region of integrons is of significant importance (Japoni‐Nejad et al. 2013). In *P. aeruginosa*, the triumvirate of Class 1, 2, and 3 integrons plays a pivotal role in conferring antibacterial resistance, resulting in MDR (Mobaraki et al. 2018). Recent studies underscore the resistance of *P. aeruginosa* isolated from clinical samples in Iran to a broad spectrum of antibiotics (Khosravi et al. 2019).

This comprehensive study delves into the molecular intricacies of Class 1, 2, and 3 integrons, exploring their dissemination and roles in antibiotic resistance via associated gene cassettes in clinical samples from various hospital wards in Qom province (central Iran) infected with *P. aeruginosa*. The identification of prevalent antibiotic resistance patterns associated with these integrons offers invaluable insights, laying the groundwork for optimal antibiotic prescribing practices that can significantly enhance patient care and expedite recovery in healthcare centers.

## 2 Materials and Methods

### 2.1 *P. aeruginosa* Bacterial Isolates

This investigation included 187 clinical isolates of *P. aeruginosa* obtained from patients aged 20–86 years. These isolates were a subset of the same collection of 200 nonduplicated clinical *P. aeruginosa* isolates previously described by Maimani et al. in a collaborative research project (Meimani et al. 2018). The original isolates were collected from four hospitals affiliated with Qom University of Medical Sciences, Iran: Shahid Beheshti, Nekoei, Kamkar, and Valiasr Hospitals. Of the original 200 isolates, 187 were included in the present study, with an approximately equal distribution among the four hospitals: 91 isolates from Shahid Beheshti Hospital, 53 from Nekoei Hospital, 32 from Kamkar Hospital, and 11 from Valiasr Hospital. The isolates were subsequently subjected to the molecular and phenotypic analyses described below. The samples included urine, blood, surgical wounds, burn wounds, feces, and general body secretions (ear, eyes, nose, and sputum), which were collected from hospitalized patients suspected of nosocomial infections and confirmed by standard microbiological assays, including microscopic, morphological, culture, and differential and biochemical diagnostic tests, and their antibiotic sensitivity and resistance patterns were determined (Table S1). The isolates, previously stored at −70°C, were thawed and allowed to reach room temperature for 2–3 h before being cultured on Brain Heart Infusion (BHI) agar (Merck, Germany). This procedure aimed to confirm the isolates and facilitate Gram staining to identify single colonies. Subsequently, Gram staining was performed on the isolated colonies, revealing Gram‐negative bacteria. Biochemical testing included the oxidase and indole tests, culture on Simmons' citrate agar, triple sugar iron (TSI) agar (Merck, Germany), and the sulfur indole motility (SIM) test. Additionally, methyl red‐Vegesproskaure (MR‐VP) tests were conducted, and pigments on the BHI agar medium were scrutinized in the colonies. Demographic data for these patients, including first and last name, gender, age, duration of hospitalization, hospital department, type of disease, and clinical sample type, were collected using structured questionnaires.

### 2.2 Drug Resistance Pattern of Isolates

The antibiotic susceptibility profile of isolates was tested by the disk diffusion method (Kirby–Bauer) according to the Clinical and Laboratory Standards Institute (CLSI) guidelines (Meimani et al. 2018). To achieve this, 18 antibiotics were classified into eight groups based on the Supporting Information (Table S2), and the pattern of drug resistance was examined for 187 clinical isolates; then, the obtained data were employed to explore the relationship between integrons and the pattern of drug resistance.

### 2.3 Identification of Samples With Class 1, 2, and 3 Integrons

#### 2.3.1 DNA Extraction

Several colonies of pure bacterial culture were removed from the BHI agar medium with a sterile loop and placed into a 1.5‐mL microtube containing 200 µL of sterile distilled water, then vortexed briefly to obtain a suspension of the colonies in distilled water. Then we placed the microtube in a bain‐marie at boiling temperature for 12 min. The mixture was then centrifuged for 10 min at 10,000 rpm. Following centrifugation, the supernatant was carefully separated using a sampler, transferred to a sterile microtube, and labeled with the corresponding sample code. This prepared supernatant was then utilized for the subsequent polymerase chain reaction (PCR).

#### 2.3.2 Quantitative DNA Analysis

The extracted genomic DNA underwent quantitative examination using a spectrophotometer. Initially, the extracted DNA was diluted by a factor of 100, and its optical absorption was measured at a wavelength of 260 nm. A nanodrop spectrophotometer (Thermo Scientific, Waltham, MA, USA) at A260/A280 nm was used to quantify the extracted genome (Hosseini Bafghi et al. 2022; Safdari et al. 2024). The extracted nucleic acid showed a 260/280 ratio of 1.85, indicating satisfactory purity and quality of the extracted genetic material (Dilhari et al. 2017; Bagheri et al. 2024).

#### 2.3.3 PCR Reaction to Check *intI1*, *intI2*, and *intI3* Genes

This work employed molecular techniques to verify the presence of three integron classes, namely, Class 1, 2, and 3 integrons, in the genomes of all 187 isolates of *P. aeruginosa*. Specific primers for these genes were designed, and their thermodynamic properties were assessed using Gene Runner version 4 software. Subsequently, the most effective primers were chosen and synthesized by the Tekaposist Company (Tehran, Iran). The primer sequences corresponding to the three genes *intI1*, *intI2*, and *intI3* are provided in Table 1.

**Table 1:** Primers were used in this study to detect Class 1, 2, and 3 integrons and identify the variable region (gene cassette) of integron Class 1.

Primer	Annealing temperature (°C)	Nucleotide sequence (5′–3′)	Amplicon size (bp)	Reference
*intI1‐*F	62	GCATCCTCGGTTTTCTGG	457	Zarei‐Yazdeli et al. (2018)
*intI1‐*R		GGTGTGGCGGGCTTCGTG		
*intI2‐*F	55	TTGCGAGTATCCATAACCTG	288	Shibata et al. (2003) and Hajiahmadi et al. (2017)
*intI2‐*R		TTACCTGCACTGGATTAAGC		
*intI3‐*F	55	ATCTGCCAAACCTGACTG	921	Kumita et al. (2009) and DI et al. (2018)
*intI3‐*R		CGAATGCCCCAACAACTC		
*intI1‐*VFP (hep58)	55	TCATGGCTTGTTATGACTGT	Variable	Zhang et al. (2020) and White et al. (2000)
*intI1‐*VRP (hep59)		GTAGGGCTTATTATGCACGC		

#### 2.3.4 Start the PCR Reaction

The amplification of genes (*intI1*, *intI2*, and *intI3*) in the *P. aeruginosa* genome was achieved by PCR using primers specific to the target genes. The materials used for the PCR assay were mixed in the microtubule in a total volume of 25 µL according to the Supporting Information (Table S3) and using the primers listed in Table 1.

The PCR reaction was executed in tandem with positive and negative controls following the provided instructions. For the *intI1* PCR reaction, the positive control comprised the extracted DNA from *Escherichia coli* strain UB1637 (pR388:In3), housing the plasmid pR388:In3 and the Class 1 integron (Maguire et al. 2001). Similarly, the *intI2* PCR reaction's positive control was *E. coli* strain DH5α (pMAQ612:Tn7), harboring plasmids pMAQ612:Tn7 and Class 2 integron (Dillon et al. 2005). The positive control for *intI3* was *E. coli* strain DH5α (pSMB731), containing the Class 3 integron (Toleman 2003). As a negative control, *E. coli* strain ATCC 25922 was utilized.

All PCR reaction components were included to monitor the reaction; however, distilled water was substituted for genomic DNA in this control setup. The PCR reactions for Class 1, 2, and 3 integrons were conducted separately, following the temperature conditions specified in the provided tables.

#### 2.3.5 PCR Product Detection and Analysis

The standard approach for visualizing and confirming PCR products involves electrophoresis on 1% agarose gel and staining with DNA Green Viewer, a safe fluorescent dye that penetrates double‐stranded DNA. After staining, the gel is placed on the transilluminator, exposing DNA bands to ultraviolet light. A photograph of the gel can then be captured. The gel includes a size indicator and the electrophoresis results for both positive and negative PCR controls. This ensures verification of both the product's precise size and the experiment's accuracy.

#### 2.3.6 Determining the Size of the Amplified Fragment in PCR

To ascertain the size of the PCR‐generated band and validate the amplification of the desired fragment, a size marker is essential. Commercially available markers exhibit a range of bands, and the selection is based on the desired fragment size. In this study, the 100‐bp plus marker from Sina Clone Company was employed, and loaded alongside the PCR products in one of the wells during electrophoresis (Figure S1).

### 2.4 PCR Reaction to Identify the Variable Region of Class 1 Integron

The variable region of Class 1 integrons was investigated in isolates that were previously confirmed to be *intI1*‐positive. PCR amplification was performed using the conserved‐region primers *intI1*‐VFP (hep58) and *intI1*‐VRP (hep59), which anneal to conserved sequences located at the 5′ and 3′ conserved regions flanking the variable region of Class 1 integrons (Table 1). Because these primers flank the variable region, the size of the resulting PCR product depends largely on the presence and number of gene cassettes inserted between the conserved regions. Accordingly, short amplification products, when present, may predominantly represent the structural/backbone portion of the integron and were not considered sufficient evidence of an informative gene‐cassette array. In contrast, larger amplicons were considered indicative of one or more inserted gene cassettes within the variable region and were selected for further molecular characterization. For amplification of the Class 1 integron variable region, Pfu DNA polymerase was used. Pfu is a high‐fidelity DNA polymerase possessing 3′ → 5′ exonuclease proofreading activity, which reduces the probability of nucleotide incorporation errors during PCR amplification. This was particularly important for the subsequent sequence‐based characterization of the integron‐associated gene‐cassette regions.

Initially, the PCR buffer was prepared by the formulation of the reaction mixture as specified in the Supporting Information (Table S3) (Hosseini Bafghi et al. 2024). Following the preparation of the reaction mixtures, the temperature program for the Class 1 integron variable‐region gene reaction was established and initiated on the thermocycler. Subsequently, the stages of detection and observation of the PCR product were carried out according to Sections 2.3.5 and 2.3.6.

### 2.5 Statistical Analysis

The results are presented as the mean and standard deviation (SD). Statistical analyses were performed using SPSS version 22. Variable differences were assessed using the Chi‐square test, while Pearson and Spearman's tests were employed to examine correlations between parameters.

## 3 Results

### 3.1 Purification of Genomic DNA

The DNA extraction from 187 *P. aeruginosa* isolates was conducted using the boiling method. Subsequently, the quality of the extracted DNA was assessed by electrophoresis on a 0.8% agarose gel.

### 3.2 The Results of Examining the *intI1* and *intI2* Genes by the PCR Method

To ascertain the presence of the Class 1 integron (*intI1* gene) in the isolates examined, DNA purified from the isolates was amplified with primers specific to this gene. As shown in Figure 1a, positive outcomes were confirmed through the visualization of a distinct 457 bp band in electrophoresis. To verify the presence of integron Class 2 (*intI2* gene) in the examined isolates, purified DNA from the isolates was added to the reaction along with primers specific to the *intI2* gene. Positive outcomes were confirmed by observing a distinct band measuring 288 bp in length during electrophoresis (Figure 1b).

**Figure 1:**
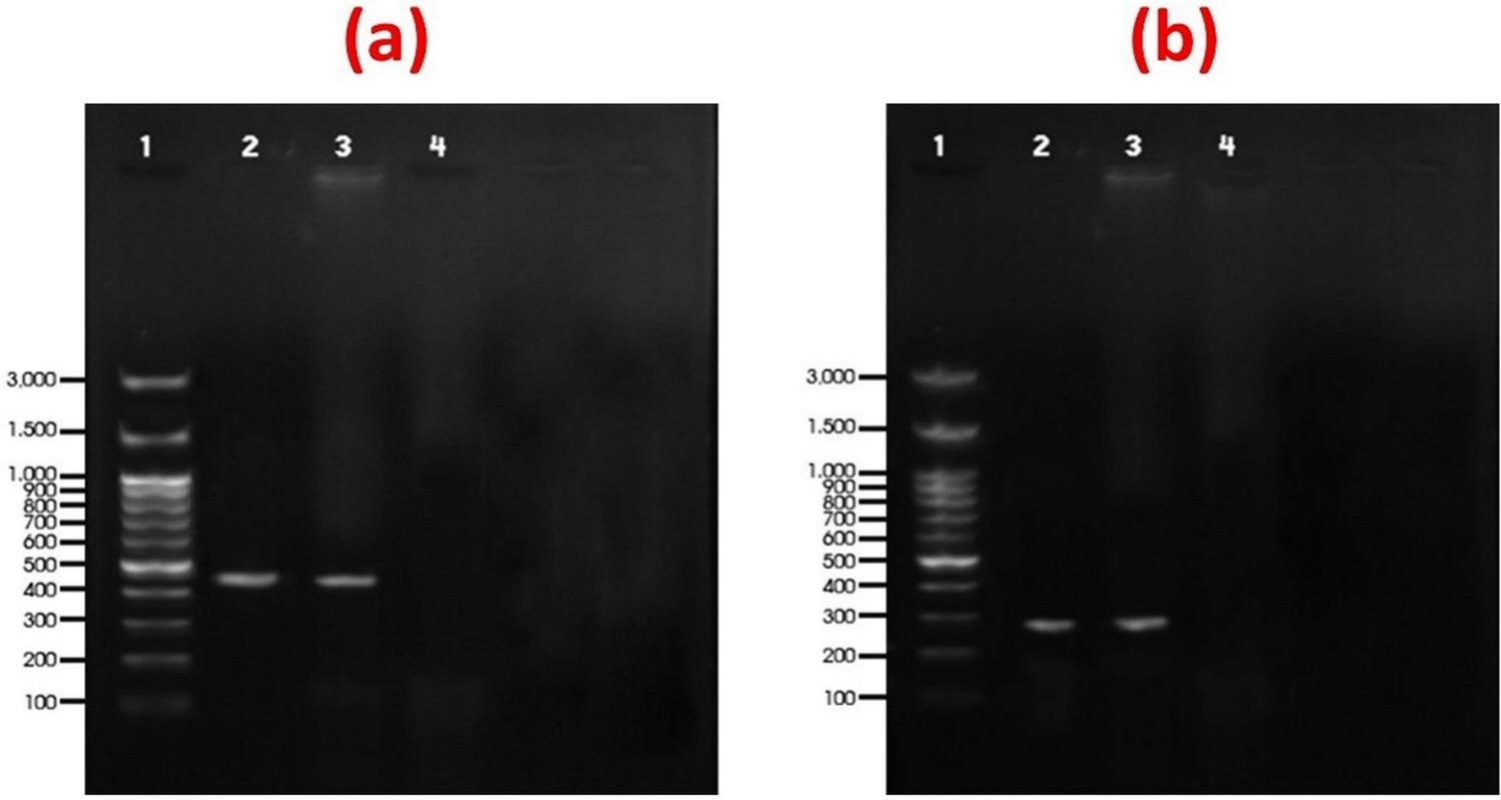
The results of *intI1* gene PCR: (1) 100 bp plus marker, (2) *intI1* positive strain, (3) *Pseudomonas aeruginosa* isolates with *intI1*, and (4) blank control (a). The results of *intI2* gene PCR: (1) 100 bp plus marker, (2) *intI2* positive strain, (3) *P. aeruginosa* isolates with *intI2*, and (4) blank control (b). PCR, polymerase chain reaction.

### 3.3 Class 1, 2, and 3 Integron Prevalence

Of the 187 clinical isolates studied, 73 had the Class 1 integron and 14 had the Class 2 integron. Therefore, the prevalence of *IntI1* and *IntI2* genes in the statistical population of this research was calculated as 39% and 7.5%, respectively (Tables S4 and S5). Of the 187 clinical isolates in this project, none were found to contain Class 3 integrons. Consequently, the prevalence of the *IntI3* gene in the statistical population of this research was zero.

### 3.4 Isolates Carrying Both *intI1* and *intI2* Genes

As shown in the Supporting Information (Table S6), among the 14 isolates harboring Class 2 integrons, 11 isolates (5.9%) were identified to possess both Class 1 and 2 integrons. These isolates were derived from samples associated with burn departments, with a frequency of five isolates (2.8%), special care, with a frequency of four isolates (2.1%), and other departments, with a frequency of two isolates (1.1%). Figure 2a,b depicts the bar graph illustrating the frequency of isolates containing both Class 1 and 2 integrons, classified by type of medical department (a) and clinical sample (b).

**Figure 2:**
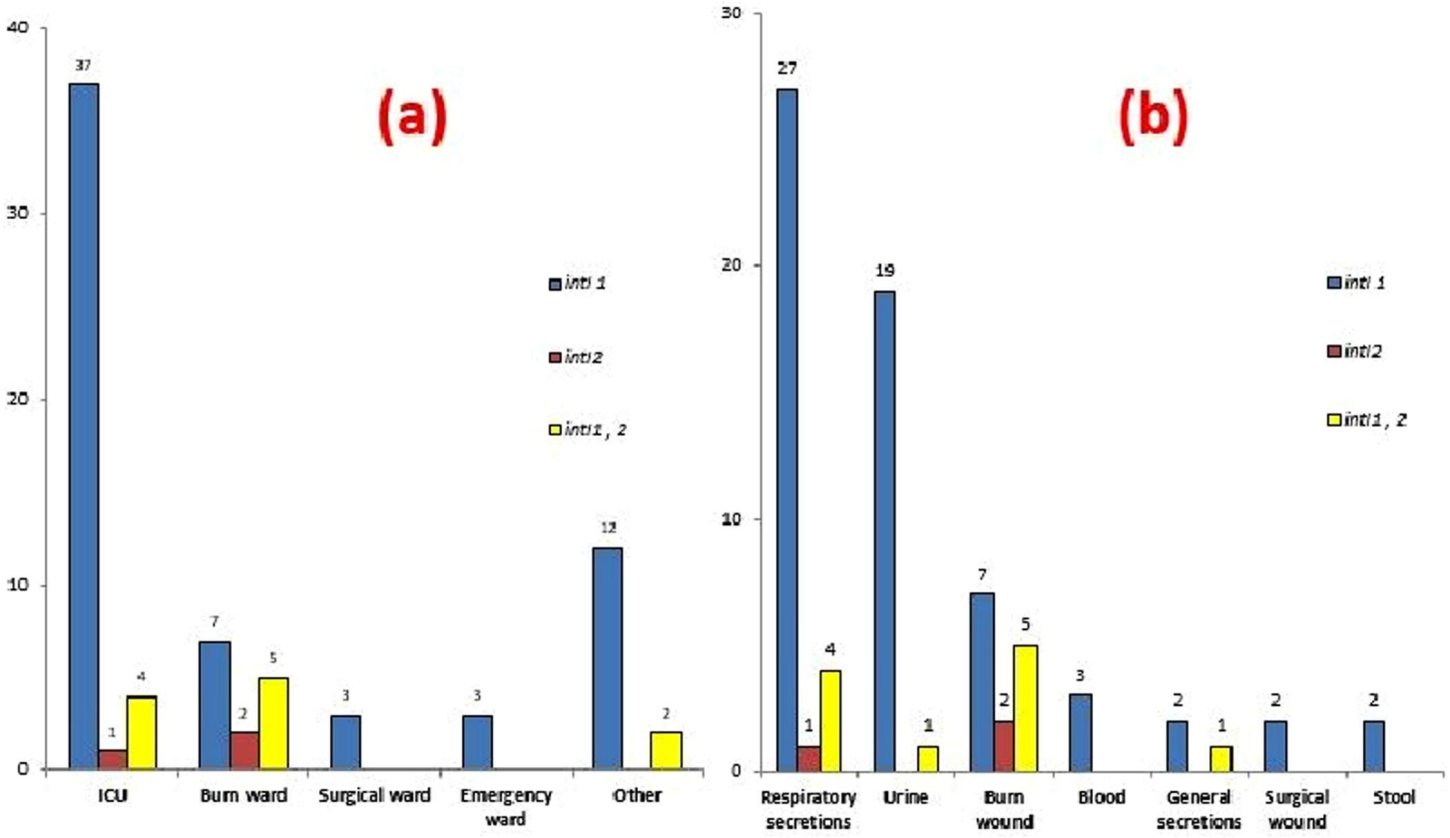
Prevalence of integron‐containing isolates (a) by medical ward and (b) by type of clinical sample.

### 3.5 Correlation of Antibiotic Resistance With Class 1 Integron Prevalence

The antibiotic resistance patterns of *P. aeruginosa* isolates to 18 selected antibiotics from various classes were examined using the Kirby–Bauer method in accordance with CLSI standards. Among the 187 clinical isolates studied, the resistance rates were as follows: 50.8% to imipenem, 51.3% to ceftazidime, 63.6% to cefotaxime, 29.4% to amikacin, 46.5% to gentamicin, 81.3% to ceftizoxime, 27.8% to piperacillin‐tazobactam, 34.8% to meropenem, 0.38% to piperacillin, and 50.3% to ceftriaxone. Additionally, resistance frequencies to ciprofloxacin, aztreonam, ticarcillin, tobramycin, cefepime, and ticarcillin‐clavulanic acid were 46.5%, 43.9%, 43.9%, 47.1%, 38.5%, and 49.2%, respectively. According to the researchers' experiments, isolates resistant to colistin and polymyxin B constituted 16.0% and 3.7% of the 187 isolates, respectively.

To explore the relationship between the presence of Class 1 integrons in clinical isolates of *P. aeruginosa* and the antibiotic resistance of the isolates, the frequency of Class 1 integron‐positive and Class 1 integron‐negative isolates categorized as resistant, intermediate, and sensitive to the 18 tested antibiotics is provided in Table 2.

**Table 2:** Antibiotic resistance of isolates of Class 1‐positive, Class 1‐negative, Class 2‐positive, and Class 2‐negative integrons obtained from antibiogram.

Antibiotic group	Antibiotics	*intI1* + (R*) *n* = 73 (39%)	*intI1* − (R*) *n* = 114 (61%)	Sum of *intI1* − and *intI1* + (R*) *n* = 187 (100%)	*IntI2* + (R*) *n* = 14 (7.5%)	*IntI2* − (R*) *n* = 173 (92.5%)	Sum of *intI2* − and *intI2* + (R*) *n* = 187 (100%)
Aminoglycosides	GEN*	38 (52%)	49 (43%)	87 (46.5%)	10 (71%)	77 (44%)	87 (46%)
	TM*	38 (52%)	50 (44%)	88 (47%)	9 (64%)	79 (45%)	88 (47%)
	AMK*	24 (33%)	31 (27%)	55 (29%)	7 (50%)	48 (28%)	55 (29%)
Antipseudomonas carbapenems	IMI*	42 (57%)	53 (46%)	95 (51%)	11 (79%)	84 (49%)	95 (51%)
	MEM*	26 (36%)	39 (36%)	65 (35%)	8 (57%)	57 (33%)	65 (35%)
Cephalosporins	CAZ*	39 (53%)	57 (50%)	96 (51%)	10 (71%)	86 (50%)	96 (51%)
	CPM*	28 (38%)	44 (39%)	72 (38%)	7 (50%)	65 (38%)	72 (38%)
	CTX*	52 (71%)	67 (59%)	119 (64%)	10 (71%)	109 (63%)	119 (64%)
	CZX*	62 (85%)	90 (79%)	152 (51%)	12 (59%)	140 (81%)	152 (81%)
	CRO*	39 (53%)	55 (48%)	94 (50%)	9 (64%)	85 (49%)	94 (50%)
Antipseudomonas fluoroquinolones	CIP*	36 (49%)	51 (45%)	87 (46%)	9 (64%)	78 (45%)	87 (46%)
Antipseudomonas penicillins + beta‐lactamase inhibitors	TCC*	36 (49%)	56 (49%)	92 (49%)	9 (64%)	83 (48%)	92 (49%)
	PTZ*	24 (33%)	28 (25%)	52 (28%)	7 (50%)	45 (26%)	52 (28%)
Antipseudomonas penicillins	PIP*	27 (37%)	44 (39%)	71 (38%)	8 (57%)	63 (36%)	71 (38%)
	TI*	33 (45%)	49 (43%)	82 (44%)	9 (64%)	73 (42%)	82 (44%)
Monobactams	ATM*	35 (48%)	47 (41%)	82 (44%)	10 (71%)	72 (42%)	82 (44%)
Polymyxins	CL*	14 (19%)	16 (14%)	30 (16%)	2 (14%)	28 (16%)	30 (16%)
	PB*	3 (4%)	4 (3%)	7 (4%)	0	7 (4%)	7 (4%)

Abbreviations: AMK, amikacin; ATM, aztreonam; CAZ, ceftazidime; CIP, ciprofloxacin; CL, Cleistin; CPM, cefepime; CRO, ceftriaxone; CTX, cefotaxime; CZX, ceftizoxime; GEN, gentamicin; IMI, imipenem; MEM, meropenem; PB, polymyxin B; PIP, piperacillin; PTZ, piperacillin‐tazobactam; R, resistant; TCC, ticarcillin‐clavulanic acid; TI, ticarcillin; TM, tobramycin.

### 3.6 Correlation of Antibiotic Resistance With Class 2 Integron Prevalence

To explore the correlation between the presence of Class 2 integrons in clinical isolates of *P. aeruginosa* and their antibiotic resistance, akin to the explanation provided for Class 1 integrons, the frequency of Class 2 integron‐positive, and Class 2 integron‐negative isolates in the categories of resistant, moderate, and sensitive to the 18 mentioned antibiotics is presented in Table 2.

### 3.7 Correlation of Resistance Pattern With Prevalence of Class 1 and 2 Integrons

#### 3.7.1 Drug Resistance Pattern for Class 1 and 2 Integrons

Drug resistance pattern, categorized by the presence and absence of Class 1 and 2 integrons, is displayed in the Supporting Information (Tables S7 and S8). In summary, among the 154 isolates classified as MDR, extensively drug‐resistant (XDR), and pan‐drug‐resistant (PDR), 66 isolates (42.9%) were found to harbor Class 1 integron.

#### 3.7.2 Chi‐Square Test for Class 1 and 2 Integrons

The desired data are subjected to a Chi‐square test to evaluate the relationship between different resistance patterns and the presence of these integrons. The probability value (*p* value) was calculated using the Chi‐square test for MDR, XDR, and PDR isolates in integrons Class 1 and 2‐positive and negative groups. The results showed that there was no significant correlation (*p* ≤ 0.05) of resistance patterns with the presence of Class 1 and 2 integrons in clinical isolates of *P. aeruginosa* (Tables S9 and S10).

### 3.8 Amplification of Class 1 Integron Gene‐Cassette Regions

The variable region of Class 1 integrons was investigated in the 73 isolates confirmed to be *intI1*‐positive. PCR was performed using the conserved‐region primers *intI1*‐VFP (hep58) and *intI1*‐VRP (hep59), which flank the variable region of the Class 1 integron.

Among the 73 *intI1*‐positive isolates, two isolates produced an approximately 800 bp amplicon, 11 produced an approximately 1700 bp amplicon, and six produced an approximately 2200 bp amplicon (Figure 3). These larger amplification products were considered informative variable‐region products because their sizes were consistent with the presence of inserted gene cassette(s) between the conserved regions of the integron. Representative products from each size category were subsequently selected for sequencing.

**Figure 3:**
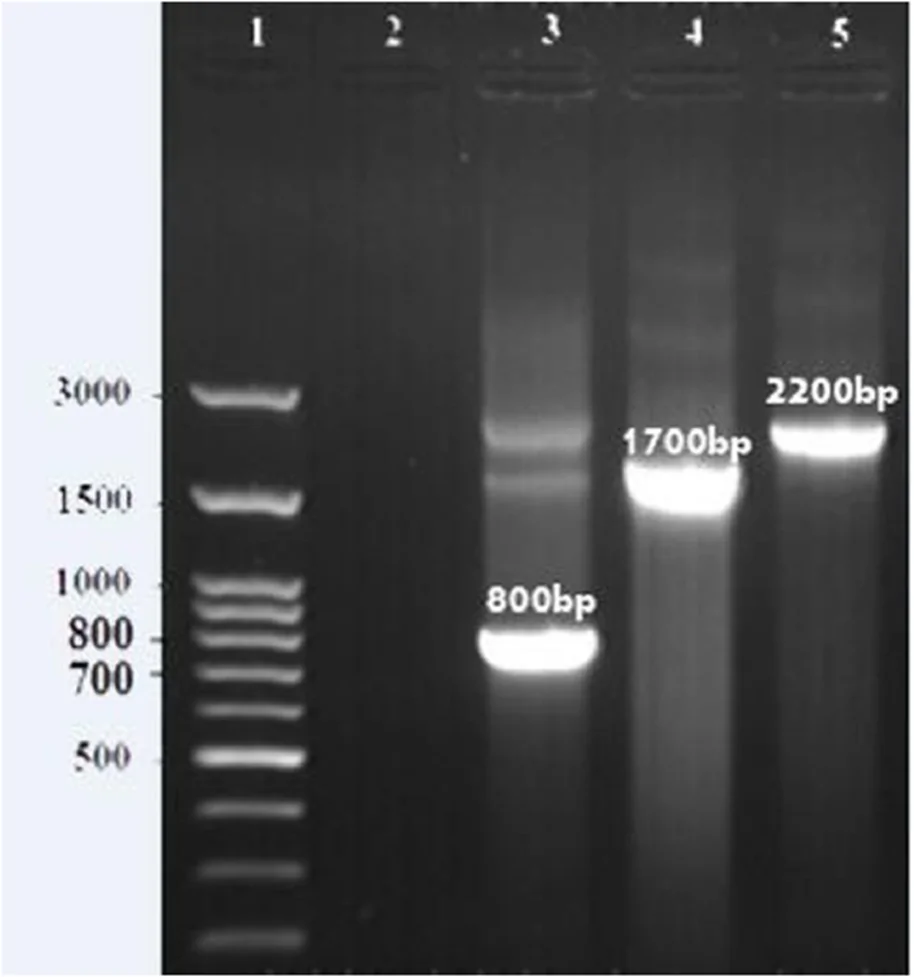
Gel electrophoresis documentation of the Class 1 integron variable region reveals the diversity of gene cassettes inserted in this region. Lane 1, Marker; Lane 2, Control Blank; Lane 3, *aadB* gene (800 bp); Lane 4, *bla*_*OXA10*_ *‐aac(6′)‐II* gene (1700 bp); Lane 5, *aadB‐bla*_*OXA10*_ *‐aac(6′)‐Ib* gene (2200 bp).

Short products resulting predominantly from the structural/backbone regions between the primer‐binding sites were not considered informative for determining the presence or composition of inserted gene cassettes. Therefore, the analysis focused on the distinct larger amplicons that indicated the presence of an extended variable region containing gene cassette(s).

The 1700 bp amplicon was the most frequently detected product, followed by the 2200 and 800 bp amplicons (Table 3). The remaining 54 *intI1*‐positive isolates did not produce a detectable informative variable‐region amplicon under the PCR conditions used. Importantly, failure to obtain such an amplicon does not necessarily demonstrate the absence of the variable region or gene cassette(s), because sequence variation at primer‐binding sites, structural variation, or other PCR‐related factors may interfere with amplification. These isolates were therefore classified as having no detectable amplification of informative variable regions rather than being definitively negative for gene cassettes.

**Table 3:** Amplification of the gene‐cassette region of Class 1 integron. The most prevalent variable regions were related to 1700 bp amplicons, while the least prevalent ones had a size of 800 bp.

Amplicon size of variable region (bp)	No. (%)	Isolation source	Prevalence
800	2 (10.5%)	Burn wound	1
		Respiratory secretions	1
1700	11 (58.0%)	Burn wound	5
		Respiratory secretions	2
		Urine	2
		Stool	1
		General Secretions	1
2200	6 (31.5%)	Burn wound	1
		Burn wound	3
		Urine	2
		Total	19

The three observed amplicon sizes were subsequently characterized by bidirectional sequencing, revealing the cassette arrangements *aadB*, *blaOXA‐10‐aac(6′)‐II*, and *aadB‐blaOXA‐10‐aac(6′)‐Ib*, respectively.

### 3.9 DNA Sequencing of the Variable Region of Class 1 Integron

Each fragment resulting from the amplification of the variable region of the Class 1 integron was selected, with one fragment from each size category chosen. The PCR product, along with the primers *intI1‐VFP* (hep58) and *intI1‐VFP* (hep58), was then used to identify the type of gene cassettes and their sequences. Sequencing was performed by Tekaposist Company, Tehran, Iran, using bidirectional reading for each of the three sequences. The outcomes are depicted in six graphs, which are presented in the Supporting Information (Figures S2–S7).

### 3.10 Analysis of DNA Sequencing Data

The DNA sequencing results for each amplified fragment from the PCR reaction were analyzed using bioinformatics software to determine the types of genes present and their respective orders. The resulting sequence was then entered into the BLAST software of the National Center for Biotechnology Information (NCBI) database to assess its similarity to sequences recorded in GenBank, and the output was thoroughly examined.

#### 3.10.1 Analyzing the 800 bp Fragment for Similarity With Sequences in the Gene Bank

Through bidirectional sequencing of the 800 bp fragment using specific primers, it was expected that the sequences obtained from the forward and reverse reads would show complementarity, either fully or predominantly. Subsequent analysis revealed that this fragment's sequence closely aligns with the coding sequence of the aminoglycoside ′2‐*O*‐adenylyl transferase *aadB* gene, as documented in the gene bank (Accession No. KX150854). The acquired data further confirm that the isolate's Class 1 integron structure includes the *intI1* gene, *the attI1* promoter, *the aadB* gene cassette, the 59‐base (59‐be) fragment, the resistance gene to quaternary ammonium compounds, sul1, and ORF5. The sequence of these gene fragments is illustrated in Figure 4.

**Figure 4:**
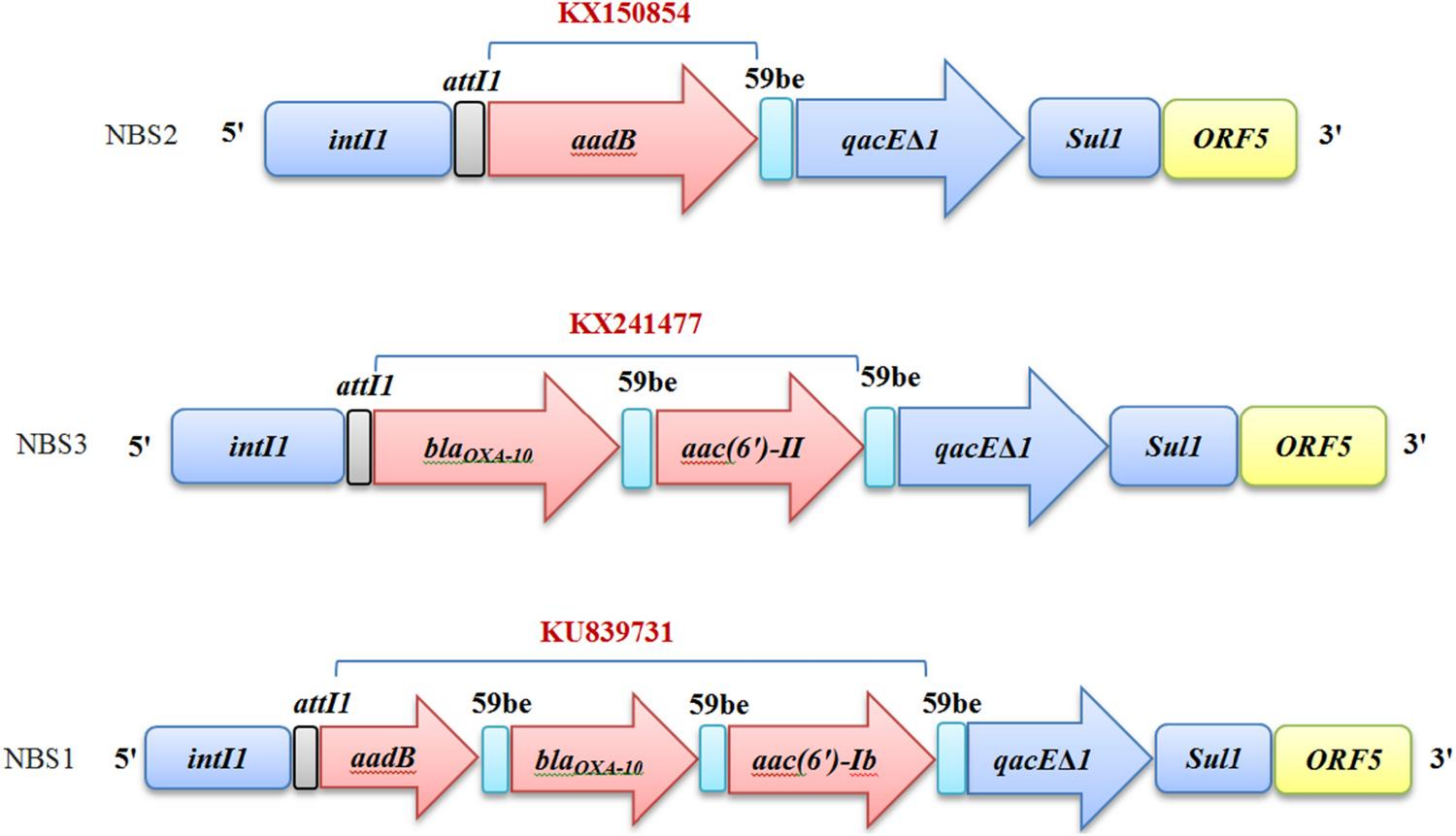
Structure of Class 1 integron as described by Levesque in 1995 (16), containing different variable regions (in red color). *intI1* gene, *attI1* promoter, gene‐cassette region, 59‐base element (59‐be), quaternary ammonium compounds and disinfectants resistance gene (*qacE*Δ*1*), sulfonamide resistance gene (*sul1*), and an open reading frame sequence (*ORF*5) are illustrated in each integron sequence within the name of the strain and the assigned accession number obtained from this study.

#### 3.10.2 1700 bp Fragment—Sequence Check in the Gene Bank

The 1700 bp fragment was read using specific primers F and R, encompassing approximately 1200 nucleotides from each side. Subsequently, the sequences were merged to generate a unified sequence.

The findings revealed that the gene cassette within the Class 1 integron fragment comprises the beta‐lactamase resistance protein *blaOXA‐10* and aminoglycoside (′6)‐*N*‐acetyltransferase *aac(6′)‐II* (*aacA4*) genes. The gene synonym for this cassette is identified as *blaOXA‐10‐aac(6′)‐II*, which is documented in the gene bank (Accession No. KX241477).

*blaOXA‐10* is classified within the group D beta‐lactamase family. Beta‐lactamases play a role in catalyzing the hydrolysis of the amide bond in the beta‐lactam ring of antibiotics from the penicillin/cephalosporin families. Within these enzymes, Class B is zinc‐containing, while Class A, C, and D enzymes operate through a serine‐based mechanism and are referred to as serine hydrolases.

On the basis of the acquired data, the Class 1 integron structure associated with this isolate comprises the *intI1* gene, the *attI1* promoter, the *blaOXA10‐aac(6′)‐II* gene cassette, the 59‐be fragment, the resistance gene to quaternary ammonium compounds, *sul1*, and ultimately ORF5. Refer to Figure 4 for the sequence representation of these gene fragments.

#### 3.10.3 Fragment 2200 bp—Checking the Nucleotide Sequence in the Gene Bank

The third fragment, derived by amplifying the Class 1 integron gene‐cassette region for nucleotide sequencing, measured 2200 bp. Sequence analysis revealed the presence of *aadB*, *blaOXA‐10*, and *aac(6′)‐Ib* resistance genes within this fragment.

On the basis of the available data, the summary of gene cassettes found in the studied Class 1 integron is as follows: aadB‐blaOXA10‐aac(6′)‐Ib, which is available in GenBank (Accession No. KU839731). This synonym is visually depicted in Figure 4.

Overall, sequencing of the three variable fragments indicated the presence of antibiotic resistance genes, including aminoglycoside adenyltransferase and aminoglycoside acetyltransferase, which are related to aminoglycoside‐modifying enzymes, along with Class D metallo‐beta‐lactamases, or oxacillinases, in clinical isolates of *P. aeruginosa*. The details of the variable regions of integron Class 1 are illustrated in Figure 4.

## 4 Discussion

The emergence of MDR bacteria is influenced by specific genes, including ARGs and integrons (An et al. 2023). The rising prevalence of these integrons leads to the incorporation of diverse gene cassettes into bacterial genomes, resulting in significant antibiotic resistance (Ghaly et al. 2020). As these cassettes proliferate in bacteria causing hospital‐acquired infections, patients face challenges with infections caused by these microorganisms, which complicate and prolong treatment as well as increase treatment costs (Mc Carlie et al. 2020). Given the varied transmission pathways and pathogenicity, understanding integron prevalence helps physicians treat infections effectively. As a result, researchers should seek to understand the underlying causes of these drug resistances in microorganisms and find specific, appropriate solutions to overcome them.

Even the prevalence of integrons and ARGs has been studied in remote sites. A study by Makowska et al. in 2020, evaluated the occurrence and frequency of clinically relevant ARGs and resistance integrons in the genomes of culturable bacteria and Class 1 integron‐integrase gene copy number in the metagenome of cryoconite, ice, and supraglacial gravel collected in the Arctic.

Our present research work examines the prevalence of Class 1, 2, and 3 integrons in MDR isolates of *P. aeruginosa and* evaluates their prevalence and expression in central hospitals in Iran.

Nikokar et al. (2013) conducted a study to inquire into the relationship between the prevalence of Class 1 integrons and MDR *P. aeruginosa* strains in hospital burn units. Their study revealed a strong correlation between the prevalence of integrons and resistance to several drugs, including imipenem, ceftazidime, piperacillin, and ciprofloxacin. The advantage of our research included the examination of Class 1, 2, and 3 integrons as well as a wider range of sample types, such as urine, blood, surgical wounds, feces, and general secretions (ear, sputum, nose, and eye) that were collected from hospitalized patients suspected of nosocomial infections.

In 2019, Shahin et al. investigated the molecular aspects of antimicrobial resistance with a focus on Class 1 and 2 integrons in *Shigella* spp. isolated from different water sources in Iran. Their research identified the potential role of ARGs and integrons in the emergence of an MDR phenotype in waterborne *Shigella* isolates.

In a cross‐sectional study in 2020, the prevalence of integrons was investigated mainly to calculate the frequency of type I, II, and III integrons in 230 isolates of MDR and methicillin‐resistant *Staphylococcus aureus* in educational hospitals of Iran, where type II and III integrons were not found in any isolates (Mohammadi et al. 2020). Similarly, in our recent research, integron 3 was not observed, but we detected integron 2 in a subset of samples with low prevalence.

In another study in 2020, Rubin et al. isolated commensal Gram‐negative bacteria from fecal samples of healthy volunteers and screened them by PCR for Class 1 integron cassette sequences and ARGs conferring resistance to ampicillin, trimethoprim‐sulfamethoxazole, gentamicin, and colistin. In our study, the antibiotic resistance pattern of MDR *P. aeruginosa* containing Class 1 integron was examined against 18 selected antibiotics across different categories.

Delarampour et al. (2020) inquired into the antibiotic resistance profiles and prevalence of Class I, II, and III integrons among 142 clinical isolates of *Klebsiella pneumoniae*, and these isolates were mostly resistant to streptomycin and ceftriaxone. Also, their results revealed that Class I integrons are widely prevalent in clinical isolates of *K. pneumoniae*, and a significant association was observed between resistance against imipenem, ciprofloxacin, gentamicin, and streptomycin and the presence of integrons, necessitating appropriate infection control programs, a finding that was corroborated in our present study.

Leungtongkam et al. (2018), in their study, pointed out two pathways of drug resistance gene transfer through plasmids and Class 1 integrons in *Acinetobacter baumannii*. They showed that, in particular, Class 1 integrons are responsible for the transfer of resistance genes such as *blaOXA‐23* and *blaPER‐1* and are identified as a factor in increasing drug resistance in environmental *Acinetobacter strains* (Leungtongkam et al. 2018). In contrast, our recent study was one of the few reports on the presence of Class 2 integron in *P. aeruginosa* species.

Another study by Japoni‐Nejad et al. on *A. baumannii* evaluated the expression and effects of Class 1 and 2 integrons and reported high expression levels leading to severe drug resistance in clinical samples (Japoni‐Nejad et al. 2013). However, our study, with a larger sample size and the inclusion of Class 3 integrons in the analysis, surpasses this study and includes analysis in *P. aeruginosa*.

Rehman et al. (2017) in a study on *E. coli* in China, found Class 1 and 2 integrons among isolates from animal and poultry food. Class 1 integrons contained gene cassettes like *dfrA1*, *dfr12*, *aadA1*, *aadA2*, *sat1*, and *orfF*. Conversely, in our work, the gene cassettes in *P. aeruginosa* Class 1 integron included *aadB*, *blaOXA10‐aac(6′)‐II*, and *aadB‐blaOXA10‐aac(6′)‐Ib*.

An important limitation of the present study is the absence of molecular typing to determine the clonal relatedness of the *P. aeruginosa* isolates. Although Class 1 and 2 integrons were detected in 73 (39.0%) and 14 (7.5%) isolates, respectively, and several integron‐associated gene‐cassette arrangements were identified, these findings alone cannot determine whether the integron‐positive isolates were genetically related or represented independent bacterial lineages. In particular, the relatively high prevalence of Class 1 integrons may reflect the circulation of resistance determinants among genetically diverse isolates, the expansion of one or more successful clones, or a combination of both mechanisms. Therefore, the present findings should not be interpreted as direct evidence of intrahospital transmission or regional clonal dissemination.

This limitation is particularly relevant because the isolates were obtained from different clinical sources and hospital departments, including burn and special‐care units. The observed distribution of integron‐positive isolates across clinical settings is descriptive and does not establish epidemiological linkage between patients or wards. Furthermore, although different integron variable‐region sizes and gene‐cassette arrangements were identified, sequencing was performed on representative fragments and these data characterize the integron‐associated resistance determinants rather than the genomic relatedness of their bacterial hosts. Thus, the current results cannot distinguish clonal expansion from horizontal acquisition and dissemination of integron‐associated resistance determinants.

Future investigations should incorporate molecular epidemiological approaches such as RAPD‐PCR, PFGE, MLST, or preferably whole‐genome sequencing to establish the genetic relatedness of isolates and to clarify whether clusters of integron‐positive *P. aeruginosa* reflect clonal transmission or independent acquisition of mobile resistance determinants. Integration of bacterial typing with temporal, patient‐level, ward‐level, and environmental sampling would provide a more robust assessment of possible transmission pathways within healthcare settings. Accordingly, the prevalence reported in the present study should be considered an estimate of the occurrence of integrons among the sampled clinical *P. aeruginosa* isolates rather than evidence of confirmed regional or intrahospital dissemination.

## 5 Conclusion

The present study demonstrates a considerable occurrence of Class 1 and 2 integrons among clinical *P. aeruginosa* isolates recovered from hospitalized patients in central Iran. The identification of integron‐associated gene cassettes carrying aminoglycoside‐ and beta‐lactam‐resistance determinants further supports the potential contribution of these mobile genetic elements to the antimicrobial resistance profile of clinical isolates. However, because molecular typing was not performed, the present study cannot determine the clonal relatedness of the isolates or establish whether the observed prevalence resulted from clonal expansion, independent acquisition, or dissemination of resistance determinants. Therefore, the findings should be interpreted as evidence of integron presence among the sampled isolates rather than as confirmation of intrahospital or regional clonal dissemination. Further molecular epidemiological studies using bacterial whole‐genome sequencing or other validated typing approaches, together with temporal and environmental sampling, are warranted to clarify transmission pathways and the epidemiological significance of integron‐positive *P. aeruginosa* in healthcare settings.

## Author Contributions

**Mahdi Hosseini Bafghi:** investigation, writing – original draft, formal analysis. **Abbas Baghaie:** software, data curation. **Razieh Nazari:** supervision, project administration, validation, data curation, writing – review and editing.

## Funding

The authors have nothing to report.

## Ethics Statement

Our study was submitted to and approved by the local ethics committee and Institutional Review Board of Islamic Azad University (IAU). This research has received ethics approval with ID: IR.IAU.QOM.REC.1402.188 issued by Islamic Azad University, Qom branch, Qom, Iran.

## Conflicts of Interest

The authors declare no conflicts of interest.

## Supporting information


Supporting File

## Data Availability

The data set generated and analyzed in this study is available on NCBI GenBank and can be accessed via the following links: https://www.ncbi.nlm.nih.gov/nuccore/1070708518, https://www.ncbi.nlm.nih.gov/nuccore/KX241477, and https://www.ncbi.nlm.nih.gov/nuccore/KU839731.
