# Supplementary material for: Dissemination of Class 1, 2, and 3 Integrons Among Multidrug‐Resistant Isolates of Pseudomonas aeruginosa
Source: Microbiologyopen. 2026 Nov 2;15(6):e70447. doi: 10.1002/mbo3.70447 (PMC13728545; doi:10.1002/mbo3.70447)
Supplement: Supplementary file 1 [file MBO3-15-e70447-s001.docx]

**Supplementary files:**

| **Supplementary Table 1. The source of isolation of the strains used in the detection of *intI1*, *inti2*, and *intI3* genes and the number of each of them according to the type of clinical sample** | |
| --- | --- |
| **Type of clinical sample** | **Number of isolates** |
| Respiratory secretions | 79 |
| Urine | 52 |
| Wounds | 30 |
| Blood | 9 |
| General secretions | 7 |
| Surgical wound | 5 |
| Wound | 3 |
| Stool | 2 |
| **Total** | **187** |

| **Supplementary Table 2. Antibiotics belonging to 8 antibiotic groups were used** | |
| --- | --- |
| **Antibiotic group** | **Name of the antibiotic** |
| Aminoglycosides | Gentamicin |
|  | Tobramycin |
|  | Amikacin |
| Anti-Pseudomonas carbapenems | Imipenem |
|  | Meropenem |
| Cephalosporins | Ceftazidime |
|  | Cefepime |
|  | Cefotaxime |
|  | Ceftizoxime |
|  | Ceftriaxone |
| Antipseudomonas fluoroquinolones | Ciprofloxacin |
| Antipseudomonas penicillins  + beta-lactamase inhibitors | Ticarcillin-clavulanic acid |
|  | Piperacillin-Tazobactam |
| Antipseudomonas penicillins | Piperacillin |
|  | Ticarcillin |
| Monobactams | Aztreonam |
| Polymyxins | Colistin |
|  | Polymyxin B |

|  |  |  | **Supplementary Table 3. PCR reaction mixture for amplification of**  ***intI1*, *intI2, intI3*,**  **and Class 1 integron gene cassettes**  **in a total volume of 25 µl** | | | |
| --- | --- | --- | --- | --- | --- | --- |
| **Required volume** | | | | **Density** |  | **Material** |
| 17.5µl | | | | 1X  1.5mM  0.2mM | PCR Buffer  Mgcl_2_  dNTP | Master mix |
| 1 µl | | | | 10 pmol/µl | Forward | Primers |
| 1 µl | | | | 10 pmol/µl | Reverse |  |
| 0.5 µl (1U) | | | |  |  | Taq DNA polymerase for Integrons  or  PFU DNA polymerase for gene cassettes |
| 5 µl | | | |  |  | DNA template |

| **Supplementary Table 4. The frequency of isolates that only contained the *intI1* gene by section and type of sample** | | | | | | | | |
| --- | --- | --- | --- | --- | --- | --- | --- | --- |
| **Gene** | **Abundance** | **Ward** | **Abundance** | **Percentage*** | **Sample type** | **Abundance** | **Percentage*** |  |
|  |  |  |  |  |  |  |  |  |
| *intI1* | 73 (39%) | Intensive care | 41 | 21.9 | Respiratory secretions | 31 | 16.6 |  |
|  |  | Burn | 12 | 6.4 | Urine | 20 | 10.7 |  |
|  |  | Surgery | 3 | 1.6 | Wounds | 12 | 6.4 |  |
|  |  | Emergency | 3 | 1.6 | Blood | 3 | 1.6 |  |
|  |  | Other | 14 | 7.5 | General secretions | 3 | 1.6 |  |
|  |  |  |  |  | Surgical wound | 2 | 1.1 |  |
|  |  |  |  |  | Stool | 2 | 1.1 |  |

| **Supplementary Table 5. The frequency of isolates that only contained the *intI2* gene by section and type of sample** | | | | | | | |
| --- | --- | --- | --- | --- | --- | --- | --- |
| **Gene** | **Abundance** | **Ward** | **Abundance** | **Percentage*** | **Sample type** | **Abundance** | **Percentage*** |
| *intI2* | 14 (7.5%) | Burn | 7 | 3.8 | Surgical wound | 7 | 3.8 |
|  |  | Intensive care | 5 | 2.8 | Respiratory secretions | 5 | 2.8 |
|  |  | Other | 2 | 1.1 | General secretions and urine | 2 | 1.1 |

* The percentages were calculated in the total number of samples (187).

| **Supplementary Table 6. The frequency of isolates that simultaneously contained the *intI1* and *intI2* genes by section and sample type** | | | | | | | |
| --- | --- | --- | --- | --- | --- | --- | --- |
| **Gene** | **Abundance** | **Ward** | **Abundance** | **Percentage*** | **Sample type** | **Abundance** | **Percentage*** |
| *intI1 & intI2* | 11 (5.9%) | Burn | 5 | 2.8 | Wounds | 5 | 2.8 |
|  |  | Intensive care | 4 | 2.1 | Respiratory secretions | 4 | 2.1 |
|  |  | Other | 2 | 1.1 | Urine | 1 | 0.53 |
|  |  |  |  |  | General secretions | 1 | 0.53 |

* The percentages were calculated in the total number of samples (187).

| **Supplementary Table 7. Illustrates the frequency of integron class 1-positive and integron class 1-negative isolates categorized by antibiotic resistance patterns, including MDR, XDR, and PDR*** | | | | |
| --- | --- | --- | --- | --- |
|  | **Number (percentage) of isolates** | | | |
|  | **MDR** | **XDR** | **PDR** | **Other isolates** |
| Integron 1  positive  n = 73 | 24 (32.9) | 40 (54.8) | 2 (2.7) | 7 (9.6) |
| Integron 1  negative  n = 114 | 40 (35.1) | 51 (44.7) | 2 (1.8) | 21 (18.4) |
| **Total (n=187)** | **64 (34.2)** | **91 (48.7)** | **4 (2.1)** | **28 (15)** |

* Antibiotic resistance pattern, according to the definition presented in **Supplementary Table 2** and using the data of the antibiogram test.

| **Supplementary Table 8. Frequency of integron class 2-positive and integron class 2-negative isolates by antibiotic resistance pattern, MDR, XDR, and PDR*** | | | | |
| --- | --- | --- | --- | --- |
|  | **Number (percentage) of isolates** | | | |
|  | **MDR** | **MDR** | **PDR** | **Other** |
| Integron 2  positive  n= 14 | 3 (21.4) | 10 (71.4) | 0 | 1 (7.1) |
| Integron 2  negative  n = 173 | 61 (35.3) | 81 (46.8) | 4 (2.3) | 27 (15.6) |
| **Total (n=187)** | **64 (34.2)** | **91 (48.7)** | **4 (2.1)** | **28 (15)** |

* Antibiotic resistance pattern, according to the definition presented in **Supplementary Table 2** and using the data of the antibiogram test.

| **Supplementary Table 9. Calculation of p-value related to chi-square test to check the dependency of antibiotic resistance pattern with the presence of class 1 integron** | | | | | | |
| --- | --- | --- | --- | --- | --- | --- |
|  | **MDR** | **Other** | **XDR** | **Other** | **PDR** | **Other** |
| Integron 1-positive | 24 | 49 | 40 | 33 | 2 | 71 |
| Integron 1-negative | 40 | 74 | 51 | 63 | 2 | 112 |
| P value* | 0.756 | | 0.179 | | 0.650 | |

*The P value was calculated using the chi-square test for MDR, XDR, and PDR isolates in integron class 1-positive and integron class 1-negative groups. The null hypothesis is the independence of antibiotic resistance patterns from having class 1 integrons. Statistical dependence is significant with P≤0.05. Therefore, according to the data, the null hypothesis is accepted.

| **Supplementary Table 10. Calculation of the p-value related to the chi-square test to check the dependence of antibiotic resistance pattern with the presence of class 2 integron** | | | | | | |
| --- | --- | --- | --- | --- | --- | --- |
|  | **MDR** | **Other** | **XDR** | **Other** | **PDR** | **Other** |
| Integron 2-positive | 3 | 11 | 10 | 4 | 0 | 14 |
| Integron 2-negative | 61 | 112 | 81 | 92 | 4 | 169 |
| P value* | 0.294 | | 0.076 | | IA* | |

*The P value was calculated using the chi-square test for MDR, XDR, and PDR isolates in integron class 2-positive and integron class 2-negative groups. The null hypothesis is the independence of antibiotic resistance patterns from having class 2 integrons. Statistical dependence is significant with P≤0.05. Therefore, according to the data, the null hypothesis is accepted. IA: It is impossible to perform the statistical test due to assigning a quantity less than 1 to one of the houses of the data table (Chi-Square approximation is probably invalid).

**Supplementary Fig. 1.** 100 bp plus marker bands.

**Supplementary Fig. 2.** Diagram of the results of 800 bp variable region sequencing with 5'CS primer

**Supplementary Fig. 3.** Diagram of the results of 800 bp variable region sequencing with 3'CS primer

**Supplementary Fig. 4.** Diagram of the results of 1700 bp variable region sequencing with 5'CS primer

**Supplementary Fig. 5.** Diagram of the results of 1700 bp variable region sequencing with 3'CS primer

**Supplementary Fig. 6.** Diagram of the results of 2200 bp variable region sequencing with 5'CS primer

**Supplementary Fig. 7.** Diagram of the results of 2200 bp variable region sequencing with 3'CS primer
